# The role of climate and out-of-Africa migration in the frequencies of risk alleles for 21 human diseases

**DOI:** 10.1186/s12863-015-0239-3

**Published:** 2015-07-14

**Authors:** Lily M. Blair, Marcus W. Feldman

**Affiliations:** Department of Biology, Stanford University, 94305 Stanford, CA USA

**Keywords:** Environmental adaptation, Human disease risk loci, Positive selection, Genome-wide association studies, Serial founder effect

## Abstract

**Background:**

Demography and environmental adaptation can affect the global distribution of genetic variants and possibly the distribution of disease. Population heterozygosity of single nucleotide polymorphisms has been shown to decrease strongly with distance from Africa and this has been attributed to the effect of serial founding events during the migration of humans out of Africa. Additionally, population allele frequencies have been shown to change due to environmental adaptation. Here, we investigate the relationship of Out-of-Africa migration and climatic variables to the distribution of risk alleles for 21 diseases.

**Results:**

For each disease, we computed the regression of average heterozygosity and average allele frequency of the risk alleles with distance from Africa and 9 environmental variables. We compared these regressions to a null distribution created by regressing statistics for SNPs not associated with disease on distance from Africa and these environmental variables. Additionally, we used Bayenv 2.0 to assess the signal of environmental adaptation associated with individual risk SNPs. For those SNPs in HGDP and HapMap that are risk alleles for type 2 diabetes, we cannot reject that their distribution is as expected from Out-of-Africa migration. However, the allelic statistics for many other diseases correlate more closely with environmental variables than would be expected from the serial founder effect and show signals of environmental adaptation. We report strong environmental interactions with several autoimmune diseases, and note a particularly strong interaction between asthma and summer humidity. Additionally, we identified several risk genes with strong environmental associations.

**Conclusions:**

For most diseases, migration does not explain the distribution of risk alleles and the worldwide pattern of allele frequencies for some diseases may be better explained by environmental associations, which suggests that some selection has acted on these diseases.

## Background

Identification of evolutionary pressures on genes associated with disease has sometimes provided information that elucidated the etiology of the disease. Heterozygote advantage for the hemoglobin A/S polymorphism in resisting the malarial parasite plasmodium falciparum is a classical example [1]. However, complex diseases are more difficult to understand, because risk for these diseases usually involves multiple loci as well as environmental factors. Genome-wide association studies (GWAS) usually find many genomic markers that are associated with disease prevalence, either increasing risk for or protection against the disease. In almost all cases such markers have small effects on the phenotype.

Although it is conceivable that such complex traits are adaptive in some environments, there is little evidence of fixed differences among populations in associated markers [2, 3]. If such adaptation were to involve weak or frequency-dependent selection for some phenotypic optimum, associated single nucleotide polymorphisms (SNPs) may not approach fixation [3, 4]. Signals of such selection that maintains these polymorphisms would be more difficult to detect than those of hard or soft sweeps.

In this paper we focus on two factors that may contribute to the distribution of genomic polymorphisms among populations: Out-of-Africa migration and environmental adaptation. Migration can have significant effects on allele frequencies; the serial founder model suggests that modern humans left Africa and began the migration through Asia to the Americas over approximately 45 k years [5–7]. When a subgroup of a population migrates to a new location, stronger genetic drift in the subgroup is expected to cause a reduction in the level of polymorphic variation. Thus, in the absence of selection, genetic heterozygosity in human populations is expected to decrease with their distance from Africa. This has been shown for microsatellite polymorphisms [8] and haplotype heterozygosities inferred from SNPs [9]. It has also been shown that local environmental variation can be associated with patterns of SNPs, suggesting that natural selection has played a role in establishing these patterns (eg. [10, 11]). Environmental variables may affect regulation of genes that contribute to phenotypic expression, and they may also influence the interaction of disease (or other) phenotypes with fitness [12]. In either case, environmental variables might affect frequency patterns of disease-associated SNPs. However, recent analyses [13, 14] have shown that populations in Africa and those outside of Africa show similar burdens of deleterious mutations, despite the differences in demographic histories experienced by these populations. This suggests that selection during the out-of-Africa migration process is not likely to have been due to differences in elimination of fitness decreasing mutations, and it raises the question of what kind of selection the environmental associations might have induced.

Here we analyze relationships to nine climate variables, including latitude, longitude, temperature, precipitation rate, humidity, and solar radiation flux. Several signals of human adaptation to these climate variables have been inferred, including in genes that are associated with cancer and immune system diseases [10, 15]. Of course, these climate variables are not the only ones that could play a role in adaptation of the disease risk loci, and other relevant variables may involve diet, subsistence type, and parasite prevalence. We do not explore the relationships of disease risk allele frequencies with these variables in this paper.

Genetic risk for some diseases has been studied in the context of migration, but these studies considered only pairwise comparisons of populations or discrete genetic differentiation events. Chen et al. [16] studied between-population differences in type 2 diabetes risk allele frequencies and showed that this genetic risk decreases from Africa to Asia to the Americas. They reported that this decrease occurred along out-of-Africa migration patterns but was more severe than would be expected by drift. Corona et al. [17] studied the effect of genetic differentiation of disease risk for 21 diseases and concluded that migration was an important factor in the worldwide pattern of genetic risk for several diseases, although this was not true of other diseases, such as biliary liver cirrhosis. They inferred the role of migration from patterns of genetic differentiation at specific branches of a genetic phylogenetic tree rather than from worldwide trends.

Berg and Coop [18] developed a method to identify polygenic adaptation using weighted risk allele frequencies of GWAS SNPs and controlling for population structure. They applied their method to three diseases and three quantitative traits and found signals of adaptation to principal components of summer and winter climate for genes related to height, skin pigmentation, ulcerative colitis, and Crohn’s disease, but not type 2 diabetes. Here we apply a similar analysis, but with a focus on the effect of migration and environmental adaptation on disease. We study 21 diseases, test each environment separately, and include two allele frequency statistics in searching for signals of selection on the risk SNPs.

It has been suggested [18–20] that differential positive selection between populations may have played a role in the distribution of disease risk alleles. We take an approach that allows us to find worldwide trends as opposed to differentiation between individual populations. Our results confirm that the frequency pattern of type 2 diabetes risk alleles has likely been determined by Out-of-Africa migration, but we also find that environmental adaptation is likely to have contributed to the worldwide frequency distributions of risk alleles for several other diseases.

## Results

### Genetic disease risk for type 2 diabetes follows Out-of-Africa migration patterns

**Fig. 1:**
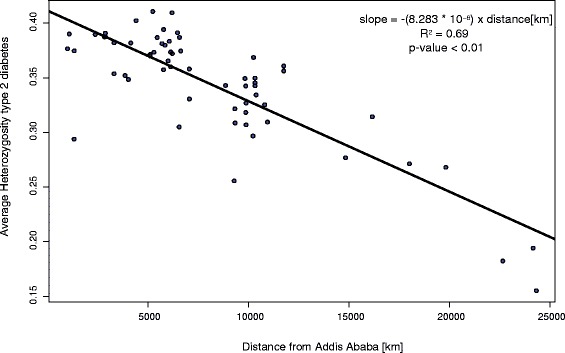
Regression of average heterozygosity of type 2 diabetes risk alleles on distance from Africa. Each point represents the average heterozygosity for one of the 61 populations studied in this paper

To determine whether the worldwide distribution of risk allele frequencies is compatible with the serial founder effect model of human migration out of Africa, we regressed average heterozygosity and average frequency of the risk allele on distance from Africa. Consistent with previous analyses [16, 17], we found that type 2 diabetes had a significant relationship with distance from Africa, with risk decreasing as distance from Africa increases; for this disease, average heterozygosity of the 15 disease risk SNPs regressed on distance from Addis Ababa had an R^2^ of 0.69 and a slope of –(8.283 * 10^^−6^)x(distance[km] from Addis Ababa), which is slightly steeper than the R^2^ and slope we calculated for average heterozygosity using all SNPs and regressed on distance from Africa (R^2^=0.62, slope = –(4.268 * 10^^−6^)). When the heterozygosity is adjusted to account for the neutral expectation (see “linear regressions” section), the R^2^ is reduced from 0.69 to 0.45 (Fig. 1). This can be compared to the R^2^ values from the regressions of the average heterozygosity of the 10,000 resampled sets of SNPs that were matched in allele frequency to the diabetes risk SNPs, on distance from Africa, and for which the mean R^2^ value was 0.33 with a standard deviation of 0.20. The empirical p-value for comparing the diabetes risk SNPs with the null distribution of resampled sets is 0.03. (see “Null Distribution” in the Methods section) However, when corrected for the tests of nine environmental variables and distance, the adjusted regression of 0.45 is not significantly different. The slope of the diabetes risk alleles on distance is steeper than expected for neutral alleles, which might suggest some type of selection against type 2 diabetes along the direction of Out-of-Africa migration, but it is very similar to the slope that Ramachandran et al. [8] report for 783 microsatellite loci (slope= -7.68*10-6) and slightly less steep than the slope Li et al. [9] report using >600,000 SNPs (slope= -1.44*10-5). The average frequency of type 2 diabetes risk alleles had an R^2^ of 0.57 for distance from Africa, and, after correction for multiple tests, was not significantly different from that obtained from random alleles. Thus, this is a borderline case, and we cannot make a strong case for selection on this disease.

### Other regressions on distance

For most diseases besides type 2 diabetes, the distribution of risk alleles did not show a strong correlation with distance from Africa. Although we found a strong relationship between heterozygosity and distance for Crohn’s disease and Parkinson’s disease (R^2^ = 0.65 and 0.57, respectively), no significant relationships between distance and heterozygosity were found for SNPs associated with any disease, including those which Corona et al. [17] suggested showed signatures of migration (Table 1). These discrepancies are likely due to the way in which genetic risk scores for each population were used by Corona et al. to construct a phylogeny of populations from which a pattern of migration was inferred by comparing the observed phylogeny with randomly generated phylogenies. In our approach, we sought an overall trend in allele frequencies that could be compared directly to expectations under the serial founder effect out of Africa. As shown by the linear relationship of heterozygosity on distance from Africa, the effect of genetic drift is constant as populations move away from Africa. Thus, by searching for a global pattern, we are able to identify genetic drift caused by sequential subsampling during the migration as opposed to genetic differentiation events that occur only between pairs of populations. We also regressed average frequency of the risk alleles on distance. Average frequency of risk alleles for type 2 diabetes, systemic sclerosis, and polycystic ovary syndrome showed the highest correlations with distance, followed by pancreatic cancer and alopecia areata.

**Table 1:** Correlation coefficient, r, between average heterozygosity and average risk allele frequency on distance from Africa, with *p*-value

Disease	SNPs	Heterozygosity r with distance	*p*-value when compared to null distribution	Risk allele frequency r with distance	*p*-value when compared to null distribution
Biliary liver cirrhosis	41	-0.04	0.03*	0.63	0.3
Alopecia areata	41	-0.42	0.78	0.69	0.04*
Prostate cancer	39	-0.22	0.33	-0.46	0.31
Systemic lupus erythematosus	33	-0.37	0.75	0.60	0.42
Ulcerative colitis	32	-0.51	0.89	-0.26	0.18
Type 1 diabetes	27	-0.23	0.74	0.23	0.89
Celiac disease	26	-0.45	0.65	-0.08	0.62
Parkinson’s disease	25	-0.76	0.33	0.16	0.99
Crohn’s disease	24	-0.81	0.10	-0.29	0.31
Membranous nephropathy	20	-0.51	0.72	-0.31	0.44
Systemic sclerosis	19	-0.69	0.41	-0.74	0.05*
Primary biliary cirrhosis	15	-0.35	0.57	-0.33	0.72
Colorectal cancer	15	-0.64	0.34	-0.66	0.05
Type 2 diabetes	15	-0.83	0.03*	-0.76	0.02*
Breast cancer	14	-0.65	0.35	-0.02	0.77
Melanoma	14	-0.28	0.51	-0.42	0.22
Rheumatoid arthritis	14	-0.24	0.64	0.43	0.56
Asthma	13	-0.10	0.46	-0.20	0.56
Neuroblastoma	10	-0.04	0.52	-0.32	0.58
Polycystic ovary syndrome	10	-0.32	0.94	0.76	0.03*
Pancreatic cancer	7	-0.32	0.95	-0.71	0.06

*P*-value was calculated by comparing the R^2^ values of the risk alleles to the null distributions created from 10,000 resampled SNP sets (see “Null Distributions” section in Methods). Correlation coefficient is reported instead of R^2^ to show directionality

*These *p*-values are not significant when Bonferroni corrected for the ten variables for each allelic statistic or when adjusted for an FDR of 0.2

### No evidence for the thrifty gene hypothesis

The thrifty gene hypothesis was proposed by Neel [21] as a possible explanation of the high prevalence of type 2 diabetes despite the potentially decreased reproductive fitness of those who have it. He argued that genes that allow for rapid and efficient metabolism of food due to an overproduction of insulin, and thus an increased risk for type 2 diabetes in the presence of certain diets, were likely to be beneficial among hunter-gatherers when food was scarce.

Under the thrifty gene hypothesis, we would expect to see positive selection of type 2 diabetes risk alleles. Because the risk alleles under positive selection would increase in frequency more than expected for neutral alleles, it is more likely that they will remain in the migrating populations. Thus average heterozygosity should not decrease in a pattern similar to that seen for microsatellites [8], which we consider “neutral”. In this study, we cannot distinguish the decrease in heterozygosity of type 2 diabetes risk alleles from the decrease that is expected due to Out-of-Africa migration. Further, under the “thrifty late” hypothesis, where the risk alleles are considered not to have been beneficial until humans migrated out of Africa [19], we would expect to see positive selection in the populations that are outside of Africa. To test these hypotheses, we ran two regressions of average heterozygosity of type 2 diabetes on distance from Africa: one excluding the 11 African populations and one excluding all populations except the 11 African populations. In the former case, the R^2^ was 0.78 with a slope of –(9.598*10^-6^) x(distance[km] from Addis Ababa), which is similar to that reported in our analysis using all populations, as well as those in Li et al.’s [9] analysis of haplotype heterozygosity using SNPs and Ramachandran et al.’s [8] analysis of microsatellites. When average heterozygosity was regressed on distance from Addis Ababa using only the African populations, the R^2^ was 0.05, which suggests a random distribution of these risk alleles in Africa. The average heterozygosity of type 2 diabetes risk alleles in Europe, Asia, and the Americas decreases with distance from Africa with a similar slope and R^2^ to that of SNPs that are not associated with disease. Although this does not indicate positive selection on these alleles, it should be stressed that these SNPs are not known to be causal for type 2 diabetes and our study does not include all SNPs known to be associated with the disease.

### Environmental variables explained more change in disease risk allelic frequency measures than distance from Africa

For many of the diseases in this study, allelic frequency measures correlated more closely with environmental variables than with distance from Africa. In particular, for neuroblastoma, the R^2^ was 0.001 for regression of average heterozygosity on distance, but was 0.58 for average heterozygosity on latitude. SNP statistics for asthma, prostate cancer, and celiac disease also showed much higher R^2^ values for regressions on a single environmental variable than for regressions on distance. To determine the variation of allele frequencies that was not due to drift, we created adjusted statistics of average risk allele heterozygosity and average risk allele frequency. For each population, we subtracted the average heterozygosity and average allele frequency using all SNPs from the average heterozygosity and average allele frequency of the risk alleles. For many diseases, the regression of these adjusted statistics on environmental variables had a higher R^2^ than the regressions of the adjusted statistics on distance from Africa.

Some of the environmental variables were correlated with each other and with distance; the absolute value of the correlations ranged from 0.09, between winter solar radiation and distance, to 0.84, between summer solar radiation and summer precipitation. The correlation of latitude and summer radiation is 0.99, and for this reason, we only included latitude in our analysis. Distance and longitude had a correlation of 0.74, because most of the Out-of-Africa migration was across longitude lines.

### Comparison with null distributions of neutral alleles showed significant relationships for many environmental variables

**Fig. 2:**
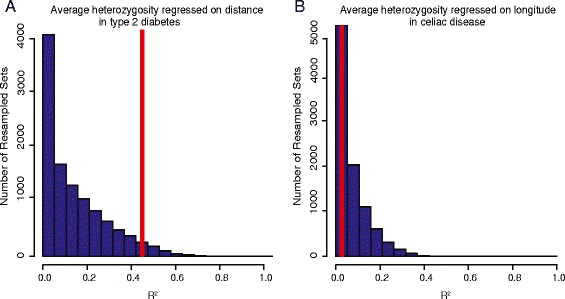
Null Distributions. Blue histograms represent the binned R^2^ values for each of 10,000 sets of resampled SNPs regressed on an environmental variable. Each resampled set contains random SNPs that match the number of risk alleles and global allele frequency of the risk alleles for that disease. Red lines indicate values of R^2^, adjusted as in Methods, with 0.45 for type 2 diabetes on distance from Africa (a) and 0.03 for celiac disease on longitude (b). Before adjustment, the R^2^ values were 0.69 for type 2 diabetes on distance from Africa and 0.13 for celiac disease on longitude. The null distributions for these two diseases are different because each null distribution is created using resampled sets that are matched for number and global allele frequency of the risk alleles. Our analysis included 15 risk alleles for type 2 diabetes and 26 for celiac disease

To determine how our regressions for disease risk SNPs compared to regressions of the same allelic measures for SNPs that were not chosen because of an association with disease, we created 10,000 sets of SNPs, matching the number of risk-associated SNPs and allele frequencies of the risk alleles. As with the disease risk alleles, we regressed adjusted average heterozygosity and adjusted average frequency of the resampled alleles on the nine environmental variables. The R^2^ of the disease risk set was compared with the R^2^ of the resampled sets and an empirical p-value was created based on the percentage of the resampled sets that had a higher R^2^ than the disease risk set (Fig. 2). Because the null distributions for each disease were created using different numbers of SNPs with different global allele frequencies, we assumed the diseases were independent of each other. We applied a Bonferroni correction for the 10 variables (nine environmental variables and distance from Africa) and the two allelic statistics. We also report significance adjusted for a false discovery rate of 0.2, which gives us more power to detect a signal. With an FDR of 0.2, we get eight significant results, and with a Bonferroni correction for the multiple environments we get four. Although we cannot infer the mechanism, our results support some selection acting on the risk alleles that has produced the observed relationship between an environmental variable and the risk allele statistics.

**Fig. 3:**
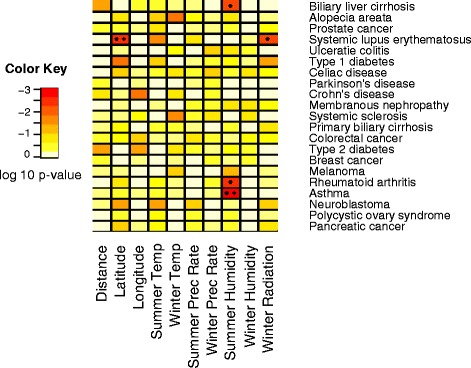
*P*-values for average heterozygosity regressed on environment. *P*-values are calculated by comparing the R^2^ of the disease risk allele heterozygosities to the null distribution created using 10,000 resampled sets of SNPs matched for number of and global allele frequency of disease risk SNPs. * Indicates significance after adjustment for an FDR of 0.2. + Indicates significance after Bonferroni correction (see “Null Distributions” section in Methods)

**Fig. 4:**
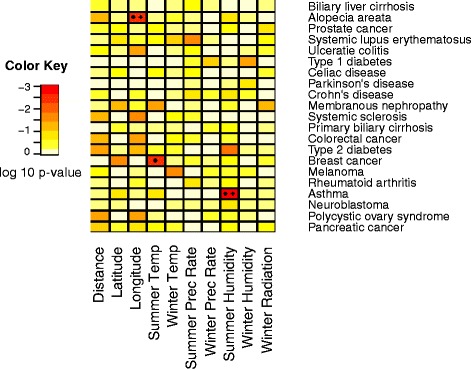
*P*-values for average risk allele frequency regressed on environment. *P*-values are calculated by comparing the R^2^ of the disease risk allele frequencies to a null distribution created using 10,000 resampled sets of SNPs matched for number of and global allele frequency of disease risk SNPs. * Indicates significance after adjustment for an FDR of 0.2. + Indicates significance after Bonferroni correction (see “Null Distributions” section in Methods)

Although correlations with distance from Africa were not significant after Bonferroni or FDR adjustments for any diseases, the R^2^ values for the disease risk allelic statistics regressed on environmental variables were significant when compared to the resampled sets for several diseases (Figs. 3 and 4). Summer humidity is significant for three diseases, and latitude, longitude, summer temperature, and winter radiation each for one. Five of the six diseases for which we report significant environmental correlations are autoimmune diseases or otherwise related to immune function. To identify functional categories that are enriched in certain environments, we ran DAVID [22, 23] to compare enrichment in the risk genes of diseases that showed significant correlation with an environmental variable to the enrichment in all disease risk genes. We did not find any significant results upon using the Bonferroni correction.

### Analysis using Bayenv shows environmental adaptation for specific SNPs and diseases

**Fig. 5:**
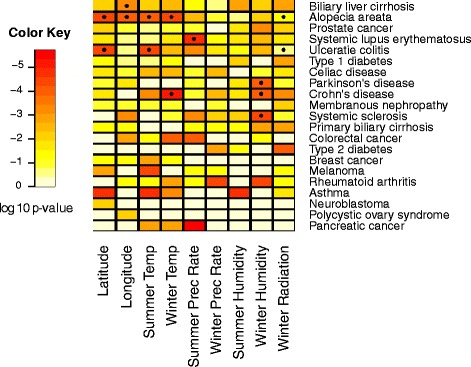
Enrichment of disease risk SNPs in the 0.05 empirical tail in Bayenv. Enrichment is indicated by color. Permutations were carried out to determine whether the number of SNPs with low *p*-values was more than expected given the total number of risk SNPs for each disease. (see “Enrichment of SNPs with low Bayenv *p*-values” section in Methods) A star indicates significance at *p*<0.05 after Bonferroni correction

We ran Bayenv 2.0 [24] to assess whether there was a signal of local environmental adaptation on the disease risk SNPs in our study. Many disease risk alleles were significant with *p*-values <0.05 in Bayenv (Fig. 5). Additionally, most of the disease/environmental variable combinations that we found to be significant in comparison to the null distributions (see Methods and “Comparison with null distributions of neutral alleles” section) had at least one risk allele that was significant in Bayenv. This confirms the relationships we found between our risk allele statistics and environmental variables.

We permuted the SNP labels to determine whether certain diseases have more SNPs that have undergone environmental adaptation than would be expected from a random set of the same number of SNPs (see Methods and “Enrichment of SNPs with low Bayenv *P*-values” section), and several diseases showed environmental adaptation. Biliary liver cirrhosis, alopecia areata, ulcerative colitis, Parkinson’s disease, Crohn’s disease, systemic sclerosis, and asthma showed significant signals of environmental adaptation for at least one environmental variable. Alopecia areata showed strong signals of adaptation for the most environmental variables, including latitude, longitude, summer temperature, winter temperature, and summer radiation. Interestingly, most of these diseases are autoimmune, which suggests there is a strong environmental effect on immune related genes or diseases.

### Identifying effects of specific environments

Berg and Coop report that Crohn’s disease and ulcerative colitis show signals of adaptation to principal components of summer and winter environmental variables. Our analysis confirms these signals and may suggest which environmental variables, as opposed to the principal components, drive this adaptation. In particular, Berg and Coop find that Crohn’s disease has a significant correlation with their summer PC2, summer PC1 and winter PC1. Summer PC2 is loaded strongly on precipitation, humidity, and radiation, and we find strong correlations for Crohn’s disease with summer precipitation and summer humidity. Similarly, summer PC1 is loaded strongly on winter temperature, which is correlated with Crohn’s disease in our analysis. Berg and Coop find that summer PC2 is also correlated with ulcerative colitis, and in our analysis ulcerative colitis is correlated with summer radiation. When compared to the null distributions, none of our correlations for Crohn’s disease or ulcerative colitis is significant after FDR or Bonferroni adjustments, but our *p*-values are similar to those that Berg and Coop report.

### Specific genes and environmental factors

Our results for the local environmental adaptation of disease risk SNPs led us to examine gene annotations of SNPs that had significant *p*-values from Bayenv as well as R^2^ values that were significantly higher than those derived from the null distributions created with sets of resampled SNPs.

Many genes that showed signals of adaptation in Bayenv [24, 25] and our analysis, and all genes that showed both signals with two or more environmental variables, had functions related to the immune system. In some cases, one SNP was associated with multiple environments and in other cases, multiple SNPs near the same gene accounted for the environmental associations. Genes that were significant with at least two variables include: BTNL2 for alopecia areata, NOD2 for Crohn’s disease, PLA2R1 for membranous neuropathy, LMO1 for neuroblastoma, and TNPO3, UBE2L3, HLA-DRA, and HIC2 for systemic lupus erythematosus.

For asthma, several genes were significant in both Bayenv and our analysis. SNPs located within 10 kb of the DENND1B gene and SNPs within 10 kb of the CRB1 gene were significant for summer humidity. The DENND1B protein is important in the innate and adaptive immune response to previously encountered antigens. It is part of the signaling pathway for inflammatory response, and is associated with moderate to severe cases of asthma [26]. SNPs within 10 kb of the RORA gene and SNPs within 10 kb of the IL2RB gene were significant for summer radiation flux. SNPs near the RORA gene were also significant for latitude, summer maximum temperature, and winter minimum temperature. RORA is a component of the mammalian circadian clock [27], and is involved in lipoprotein metabolism [28] and lipid homeostasis in muscle cells [29]. These functions could help explain the signals of adaptation of this asthma-associated gene with summer and winter temperature.

For prostate cancer, SNPs located within 10 kb the KLK3 gene were significant for summer radiation flux, winter humidity, and latitude in Bayenv. The KLK3 gene produces prostate specific antigen (PSA), a widely used biomarker for prostate cancer [30].

## Discussion

We have explored the effect of Out-of-Africa migration and climate variables on allele frequency statistics for risk SNPs associated with 21 diseases. To compare the effects on risk allele frequency statistics of Out-of-Africa migration and environmental selection, we regressed average heterozygosity and average risk allele frequency on distance from Addis Ababa, which served as a proxy for Out-of-Africa migration, and on nine environmental variables. To determine significance, we compared these regressions to those from 10,000 random samples. In addition, we used the Bayenv program to search for signals of selection that could reflect environmental adaptation of these risk allele SNPs. Although some methods weight the risk alleles by effect size (eg. [18]), we choose not to do so, as the effect sizes are often measured in one population and it is not clear that a SNP would have the same effect size in other populations.

Type 2 diabetes showed the highest correlation with accepted patterns of human migration. Average heterozygosity of the 15 risk alleles decreased with distance from Africa with an R^2^ of 0.69, which is similar to the regression found by Ramachandran et al. [8] using microsatellite loci and the regression found by Li et al. [9] using haplotype heterozygosity on Chromosome 19. This pattern of risk allele frequencies may be due to the risk of type 2 diabetes increasing greatly with specific dietary customs that most probably spread after the major Out-of-Africa migrations. In that scenario, the risk alleles should be distributed similarly to neutral alleles, and because the linear relation between the average heterozygosity of the type 2 diabetes risk alleles and distance from Africa is similar to that of large sets of SNPs and microsatellite loci, this explanation seems plausible.

There could be selection against other SNPs that have not yet been associated with type 2 diabetes or are not included in our data set. These SNPs have the potential to increase or decrease the correlation of heterozygosity and risk allele frequency with distance from Africa and might contribute to selection along migratory paths. It has previously been reported that type 2 diabetes risk alleles are more genetically differentiated across populations than expected by chance [16]. Chen et al. hypothesize that this shift in allele frequencies may be due to the development of agricultural methods at different times in different populations, the thrifty gene hypothesis, or an evolutionary pressure caused by the mismatch between genetics and the available diet, which could occur when populations migrate. We see no evidence for the “thrifty late” hypothesis, as average heterozygosity of type 2 diabetes risk alleles among populations outside of Africa shows a regression similar to that of all populations including Africa. Another hypothesis, the “drifty gene” hypothesis, suggests that genes that are associated with type 2 diabetes may have been subject to drift in human populations as the selection against obesity was relaxed with the release from predatory pressure [31]. Our results are more consistent with this hypothesis. In light of the lack of signals of environmental adaptation for the risk alleles of this disease and the similar geographic pattern of these risk allele statistics to that of the whole HGDP and HapMap data set, we conclude that migration was probably important in establishing the world wide-pattern of type 2 diabetes risk allele frequencies. Our analysis suggests that any selection on the type 2 diabetes risk alleles analyzed in this study must have been weak at most.

For most diseases in this study, however, allele frequency measures did not correlate strongly with distance from Africa, which suggests that for these diseases factors other than migration have affected the distribution of risk alleles. Corona et al. [17] use genetic differentiation between populations at individual branches of a genetic phylogenetic tree to suggest that risks for several diseases are associated with migration, but we conclude here that this genetic differentiation between populations on one side of a phylogenetic tree and populations on the other is not representative of an overall pattern of allele frequencies that would have been due to the serial founder effect during the Out-of-Africa migration.

Many of the diseases studied here show signals of adaptation to one or more environmental variables, and the relationship between environment and risk allele frequency measures is much stronger than with distance from Africa. In particular, we identified strong environmental interactions with asthma risk SNPs near the DENND1B and RORA genes and strong environmental interactions with alopecia areata, Crohn’s disease, membranous neuropathy, neuroblastoma, and systemic lupus erythematosus in several immune system genes. These gene-environment interactions are inferred from the R^2^ of risk allele frequency measures compared to those obtained from null distributions as well as from Bayenv, and may explain patterns of allele statistics for these diseases. Additionally, summer humidity appears to have a particularly strong interaction with asthma, as well as rheumatoid arthritis and biliary liver cirrhosis, suggesting that some form of selection on these diseases could have produced the pattern of environmental associations described here. We conclude that the signal from environmental variables is stronger than that from migration for many diseases, particularly those related to immune system function and including those that Corona et al. [17] suggested were determined by migration patterns. Therefore, genetic differentiation between individual branches of the phylogenetic tree may not be sufficient to infer that migration patterns explain the worldwide distribution of risk alleles. We reported signals of environmental adaptation that, for the three diseases in our study and theirs, are similar to what Berg and Coop [18] reported, and correlations with specific environmental variables that may explain the signals of adaptation they inferred from principal components of summer and winter climates.

It is likely that changes in population allele frequencies in response to environmental variables have occurred over long time spans. However, as it is difficult to estimate the history of climate variables, we used current climate data as a proxy for the environmental pressures that may have caused selection on risk alleles. Because the regressions of allele frequencies on environmental variables depend on the differences in climate variables between populations rather absolute values, we expect that current climate data are sufficient for our analyses. It is also possible, however, that there have been historical environmental changes not encompassed by the variables we used that have affected the worldwide frequencies of risk alleles.

## Conclusions

In this study, we infer signals of environmental adaptation for several diseases and disease risk genes, particularly those related to immune system function, and suggest that climate has had a stronger effect on disease risk allele frequencies for some diseases than Out-of-Africa migration. Our analysis, as in all studies using GWAS SNPs, has limitations, including the possibility of missing variants and incorrect estimation of causal sites, particularly in populations in which the GWA studies were not conducted; thus we should be careful in interpreting the signals of adaptation reported here, and we cannot identify the mechanism(s) of selection that may have acted on these SNPs and diseases. Additionally, we note that disease prevalence can change rapidly without genetic changes, such as the recent increases of type 2 diabetes in China and India that are likely due to non-gene related change in behavior. Thus the environmental associations reported here cannot be used as a prediction of disease in certain populations or locations. That said, our analysis suggests that we cannot discount the effect of environment on the frequencies of risk loci for the diseases studied here.

## Methods

### Disease risk SNPs

Disease risk SNPs and disease likelihoods were gathered from [17] for all diseases with at least 14 risk alleles that are polymorphic in the HGDP [9] and HapMap [32] SNP data sets. We also included asthma, neuroblastoma, polycystic ovary syndrome, and pancreatic cancer, which had 13, 10, 10, and 7 disease risk alleles, respectively, and were observed by Corona et al to show some genetic risk differentiation among populations. From the patterns of disease risk variation, the level of dependence on migration of the geographical variation in disease risk was inferred. All disease risk SNPs used were detected in at least two populations and had a p-value less than 10^-6^ for association with disease. Additionally, SNPs that were in linkage disequilibrium (R^2^≥0.2) were removed to leave one SNP per region [17].

In the present study, population allele frequencies of these disease risk SNPs used 564,201 SNPs from samples of 52 Human Genome Diversity Panel (HGDP) populations and 9 HapMap populations (all except MEX and ASW) presented by Pemberton et al. [33]. SNPs that were not present in both data sets were excluded from this analysis.

### Distance from Africa

Distance from Africa for each population was calculated as in [8] using the great circle distance with waypoints. The origin was Addis Ababa, Ethiopia, and waypoints were Anadyr, Russia; Cairo, Egypt; Istanbul, Turkey; Phnom Penh, Cambodia; and Prince Rupert, Canada. Total distances were calculated as the sum of the great circle distance from the origin to the connecting waypoint plus the great circle distance from the waypoint to the sample location, plus the great circle distance between the waypoints if two or more connecting waypoints were needed. Geographical locations of the samples were reported in [34] for HGDP populations and [33] for HapMap populations.

### Environmental variables

Climate data for each population included nine variables: latitude, longitude, minimum winter temperature, maximum summer temperature, winter precipitation rate, summer precipitation rate, winter radiation flux, winter humidity, and summer humidity. Monthly averages of these variables from 1982-2013 were obtained from NCEP-DOE Reanalysis database [35]. Data were averaged over all years and over June, July, and August for summer variables, and December, January, and February for the winter variables. Because latitude and summer radiation flux had a correlation coefficient of 0.99, we included only latitude in our analysis.

### Linear regressions

For the disease risk SNPs, average heterozygosity and average frequency of the risk allele were regressed on distance from Africa and each of the nine climate variables as a reflection of drift or selection. For each disease, we define average heterozygosity as${\displaystyle \sum_{i=1}^d2{p}_{ij}{q}_{ij}}/d$ and frequency of the risk allele as ${\displaystyle \sum_{i=1}^d{p}_{ij}}/d$, where *p*_*ij*_ is the sample allele frequency of the risk allele *i* in population *j*, *q*_*ij*_ =1- *p*_*ij*_, and *d* is the number of risk alleles for a disease. We regressed these two statistics for each disease on distance from Africa, which serves as a proxy for the effects of migration and drift [8, 9].

Next, we were interested in inferring which environmental variables could explain the variance in allele frequencies not accounted for by drift. For each population, we calculated the average heterozygosity and average allele frequencies using all 564,201 SNPs, and then subtracted that from the average heterozygosity of the risk alleles and average risk allele frequency, respectively. Because allelic statistics averaged over all SNPs should be representative of population structure due to migration out of Africa, this subtraction leaves us with the variance not accounted for by drift. We regressed these adjusted allelic statistics on the nine environmental variables, separately. To test whether unequal error variances affected our allele frequency regressions, we calculated the variance of the average risk allele frequencies for each population and carried out regressions on each environmental variable using a weighted linear model, where the weights were the inverses of the population variances. We calculated the significance of these regressions using null distributions created from weighted regressions of the 10,000 resampled sets of alleles. The results were consistent with the original results. That is, the environmental correlations that were significant after correction for multiple tests using the unweighted linear regression were still significant after correction for multiple tests using the weighted regression, and no significant relationships were found using the weighted regression that were not found using the unweighted linear regression (Results not shown).

### Null distributions

For each disease, we created 10,000 sets of HGDP and HapMap SNPs from [33] that matched the set of risk SNPs in number of SNPs and global allele frequencies. All HGDP and HapMap SNPs were grouped into bins of 0.1 by global allele frequency. To create one resampled set for a disease, each risk SNP was replaced by a random SNP with a global allele frequency (averaged over all populations) in the same bin as the global allele frequency of the risk allele. For example, if disease A has two risk alleles, rs1 and rs2, and these risk SNPs have global allele frequencies of 0.16 and 0.34, respectively, then a resampled set for this disease might have rs5 and rs6 with global allele frequencies of 0.18 and 0.31, respectively. In each HGDP and HapMap population, allele frequencies at the SNPs that were randomly chosen according to the disease and global frequencies were calculated from the [33] dataset and then used to produce the adjusted average heterozygosity and adjusted average frequency (see “Linear regressions” section) of the resampled SNPs. These two statistics were then regressed on distance and the environmental variables, and an R^2^ value was computed. The process was repeated 10,000 times for each disease. For each allelic statistic for each disease, histograms of R^2^ for the 10,000 resampled sets of SNPs were made for all variables, and these histograms functioned as null distributions. Then the estimated R^2^ for each of the disease risk allele statistics was compared to the appropriate null distribution of resampled R^2^ values. Because we produce the distributions for each disease using a different number of SNPs and SNPs with different global frequencies, we assume the null distributions for each disease are independent from one another. But, although the null distributions are different for each environment as well, we cannot assume these are independent from one another as they are created from the same resampled set of SNPs. Thus, we use a Bonferroni correction for the ten tested variables (nine environmental variables and distance from Africa) and the two allelic statistics, and if the R^2^ for the disease risk allele statistics is in the top 0.0025 % of the latter, we infer that the variable had some effect on the risk allele frequencies. Because the environmental variables are correlated we also adjusted the *p*-values using a false discovery rate of 0.2, which gave us more power to detect effects.

We also made resampled sets by matching the resampled alleles to the average European allele frequency of the risk SNP, because GWA studies are often done in European populations. This analysis produced similar results.

### Bayenv runs

Bayenv 2.0 ([24, 25]; see also [36]) was run using 564,201 SNPs from samples of 52 HGDP and 9 HapMap populations [33]. Samples had been genotyped on the Illumina 650 K array, which includes three ascertainment panels (250 K, 300 K, and AFR). For each ascertainment panel, a covariance matrix between the populations was calculated using 10,000 randomly sampled alleles and 100,000 MCMC iterations. Then two runs per ascertainment panel were carried out, each using 1,000,000 MCMC iterations, to determine the Bayes Factor for each SNP in association with each of the nine environmental variables included in this study. Bayes factors were averaged over the two runs, ascertainment panels were combined into one file, and ranked *p*-values were calculated. These Bayes factors represent the magnitude of environmental adaptation of a single SNP, but because they are dependent on the null model and may be inflated due to imperfections in the model, ranked *p*-values provide a more conservative estimate of the signal of selection. As in [10], we considered empirical *p*-values produced from the Bayenv analysis that are less than 0.05 to be significant and to be indicative of signals of environmental adaptation for specific alleles. This serves as confirmation of the high correlations of some of the risk alleles and environmental variables reported in the results.

### Enrichment of SNPs with low Bayenv *p*-values

Enrichment of disease risk SNPs in the tail of the Bayenv empirical *p*-values was calculated with the following equation:

$$ \mathrm{enrichment}=\frac{\frac{{\mathrm{n}}_{\mathrm{r}}}{{\mathrm{n}}_{\mathrm{n}\mathrm{r}}}}{\frac{{\mathrm{N}}_{\mathrm{r}}}{{\mathrm{N}}_{\mathrm{n}\mathrm{r}}}}, $$

where n_r_ and n_nr_ are the number of risk SNPs and SNPs not associated with disease, respectively, in the 0.05 empirical tail, and N_r_ and N_nr_ are the number of risk SNPs and SNPs not associated with disease, respectively, among all tested SNPs.

To determine whether the number of risk SNPs that had *p*-values less than 0.05 in Bayenv was more than expected, given the total number of risk SNPs for a disease, for each disease, we created 50,000 sets of random SNPs that matched the total number of disease risk SNPs. We then calculated how often the sets of random SNPs contained more SNPs with low *p*-values than the disease risk SNPs, for each variable. We applied a Bonferroni correction for the multiple tests, so our cutoff for significance was *p*=0.00026.

### Gene annotations

A SNP was considered genic if it was within 10 kb of a gene. Gene locations were obtained from University of California, Santa Cruz (UCSC) refFlat mappings of RefSeq genes to hg18 (Build 36) (downloaded in March 2013, http://www.genome.ucsc.edu)

## Abbreviations

SNP: Single nucleotide polymorphism
GWAS: Genome-wide association studies
HGDP: Human Genome Diversity Project
